# The Myokine Adaptome in Health and Disease: Exercise-Induced Cellular Signaling, Muscle–Organ Crosstalk, and Therapeutic Plasticity

**DOI:** 10.3390/cells15141236

**Published:** 2026-07-09

**Authors:** Dan Cristian Mănescu, Camelia Daniela Plastoi, Ancuța Pîrvan, Rodica Dîrnu, Elena Ancuța Floroiu, Andreea Popescu

**Affiliations:** 1 Department of Physical Education and Sports, Bucharest University of Economic Sciences, 010374 Bucharest, Romania; dan.manescu@defs.ase.ro; 2 Sport and Health Department, Faculty of Medical and Behavioral Sciences, Constantin Brâncusi University of Târgu-Jiu, 210135 Targu-Jiu, Romania; rodica.dirnu@e-ucb.ro (R.D.); andreea.popescu@e-ucb.ro (A.P.); 3 Department of Physical Education and Sports, Faculty of Humanities, Valahia University of Târgoviste, 130105 Targoviste, Romania; 4 Doctoral School in Sport Science and Physical Education, Pitești University Center, National University of Science and Technology Politehnica Bucharest, 110040 Pitesti, Romania; floroiu.ancuta@yahoo.com

**Keywords:** myokines, skeletal muscle, exercise, muscle–organ crosstalk, exerkines, extracellular vesicles, AMPK, mTORC1, inflammation, sarcop chronic disease, exercise medicine

## Abstract

**Highlights:**

**What are the main findings?**

- Skeletal muscle is conceptualized as a context-sensitive signal-processing organ that integrates energetic, mechanical, calcium-dependent, redox and inflammatory cues into coordinated myokine and exerkine signatures.
- The review proposes the “myokine adaptome” as a testable framework describing how acute exercise, chronic training, aging and disease reshape muscle–organ communication across amplitude, timing, molecular composition and target-organ sensitivity.
- The adaptome is defined as an operational information-processing framework rather than a molecular inventory, requiring source confidence, temporal resolution, co-signal context and functional decoding to be evaluated together.

**What are the implications of the main findings?**

- Myokine biology offers a mechanistic bridge between exercise, metabolic regulation, inflammation, sarcopenia, osteoporosis, COPD, cardiovascular disease, cancer/cachexia and neuroplasticity.
- Time-resolved, source-informed and panel-based profiling, combined with explicitly testable predictions, may support biomarker discovery, rehabilitation monitoring and personalized exercise medicine without reducing the benefits of exercise to a single molecule.

**Abstract:**

Skeletal muscle is increasingly recognized as a dynamic secretory organ capable of translating contractile, metabolic, mechanical and inflammatory stimuli into systemic biological signals. Among these signals, myokines and myokine-associated exerkines mediate communication between skeletal muscle and distant organs, influencing glucose and lipid metabolism, immune regulation, bone remodeling, neuroplasticity, vascular function and tissue regeneration. Representative mediators considered include IL-6, IL-15, myostatin, follistatin, decorin, FNDC5/irisin, FGF21, myonectin/CTRP15, BDNF, cathepsin B, SPARC, apelin and extracellular-vesicle cargo. However, current evidence remains fragmented across individual molecules, exercise modalities, sampling windows, assay platforms and disease contexts. This narrative mechanistic review proposes the concept of the “myokine adaptome” as an integrated, context-dependent signaling network through which skeletal muscle contributes to systemic homeostasis in health and disease. We synthesize evidence on cellular triggers of myokine release, including AMPK-PGC-1α signaling, mTORC1-dependent mechanical sensing, calcium flux, redox signaling, inflammatory pathways and extracellular-vesicle-mediated communication. We further examine how exercise modality, aging, obesity, type 2 diabetes, sarcopenia, osteoporosis, cardiovascular disease, COPD, cancer/cachexia and chronic inflammation reshape myokine production and target-organ responsiveness. The central argument is that myokine biology should be interpreted not as a catalog of isolated mediators, but as a dynamic adaptive code defined by signal amplitude, temporal pattern, molecular composition, delivery route and recipient-tissue sensitivity. Its novelty is operational rather than nominal: it requires source confidence, temporal kinetics, co-signal context, delivery route and functional decoding to be evaluated together. This framework may improve biomarker design, disease-specific exercise prescription and therapeutic strategies aimed at restoring adaptive muscle–organ communication. The framework is further strengthened by testable predictions concerning adaptive pulsatility, modality-specific signatures, source attribution, recovery quality, disease-specific decoding and the superiority of multi-marker panels over single-molecule readouts.

## 1. Introduction

Skeletal muscle has traditionally been interpreted through the language of force production, locomotion and energy expenditure. This view has been substantially expanded by the recognition that contracting muscle releases cytokines, peptides, growth factors, metabolites and vesicle-associated cargo with local and systemic biological effects. The concept of skeletal muscle as a secretory organ has therefore become central to understanding how physical activity protects against metabolic, inflammatory, musculoskeletal, cardiopulmonary and neurodegenerative disease [1,2,3].

The term myokine was originally used to describe cytokines and other peptides produced and released by muscle fibers with autocrine, paracrine or endocrine effects. The field has since broadened to include muscle-derived growth factors, extracellular-matrix-associated mediators, metabokines, lipokines and extracellular-vesicle cargo. This expansion has improved biological coverage but has also introduced conceptual ambiguity, because many circulating exercise-responsive molecules are not exclusively produced by skeletal muscle and may instead reflect coordinated responses from adipose tissue, liver, immune cells, endothelium or bone [4,5,6,7].

Exercise biology is consequently moving from a molecule-by-molecule description of the muscle secretome toward systems-level models in which contraction is viewed as a complex cellular perturbation. A single bout of exercise simultaneously changes ATP turnover, AMP/ADP ratios, calcium flux, redox tone, glycogen availability, mechanical strain, temperature, pH, perfusion and immune-cell trafficking. These inputs converge on transcriptional, translational and post-translational networks that determine which signals are released, when they appear, how long they persist and which tissues can decode them [8,9,10,11].

Recent conceptual work has framed the skeletal muscle secretome as an integrated endocrine code defined by secretion kinetics, co-released molecular combinations, delivery modalities and target-tissue receptor landscapes [6]. The present review builds on this logic but emphasizes a complementary adaptation-centered perspective. We propose the term myokine adaptome to describe the dynamic, context-dependent signaling network through which skeletal muscle translates energetic, mechanical, redox, calcium-dependent and inflammatory information into systemic metabolic, immune, osteogenic, neurovascular and regenerative outcomes.

The review is organized around five guiding questions:

**Q1.** Which intracellular sensors and cellular stress inputs determine the release of myokines, myokine-associated exerkines and extracellular-vesicle cargo during exercise?
**Q2.** How do exercise modality, dose, timing and training status shape the amplitude, temporal pattern and molecular composition of the myokine response?
**Q3.** How do aging, obesity, type 2 diabetes, sarcopenia, osteoporosis, cardiovascular disease, COPD, cancer/cachexia and chronic inflammation distort adaptive muscle–organ communication?
**Q4.** How can the myokine adaptome be distinguished from the broader muscle secretome and from non-muscle exerkine responses in a biologically rigorous way?
**Q5.** How can time-resolved myokine profiling be translated into biomarkers, rehabilitation monitoring and personalized exercise medicine without overclaiming single-molecule causality?

By addressing these questions, this review makes a conceptual and translational contribution: it reframes myokine biology from a static catalog of molecules into a testable model of adaptive communication. The goal is not to rename the muscle secretome, but to provide a disciplined framework for interpreting when a signal is muscle-derived, when it is exercise-responsive but not muscle-exclusive, when it is adaptive or maladaptive, and when it is clinically actionable. This approach is intended primarily to help researchers design better time-resolved studies and, after validation, may help clinicians interpret exercise responses in disease-specific contexts and support the development of personalized exercise prescriptions that restore coordinated muscle–organ communication rather than targeting isolated mediators one by one.

To clarify the added value of the framework, the adaptome is not introduced as a new class of molecules or as a replacement for the muscle secretome. It is proposed as an operational interpretive layer that requires four conditions to be considered together: source confidence, temporal architecture, co-signal composition and target-organ decoding linked to functional outcomes. Secretome studies primarily ask what is released, exerkine studies ask what changes with exercise, and inter-organ crosstalk studies ask which tissues communicate; the adaptome asks whether the generated pattern carries adaptive information and how that claim can be weakened or falsified [4,5,6,7,12,13,14,15,16,17,18,19,20].

The proposed model is organized around five interacting layers rather than introduced as a fixed biomarker panel: contractile input, energetic state, mechanical state, inflammatory-redox state and recipient-tissue state. Together, these layers specify how exercise dose is converted into a biological message, how fuel availability and loading history shape secretory output, how acute inflammatory-redox signals resolve or become maladaptive, and how target organs determine whether a circulating signal becomes functional adaptation [1,2,3,4,5,6,7,8,9,10,11,12,13,14,15,16,17].

## 2. Narrative Review Methodology

This article is a narrative mechanistic review rather than a systematic review or meta-analysis. The aim was to synthesize mechanistic, translational and clinical evidence into an integrative framework for understanding exercise-induced muscle–organ communication. The literature was mapped through targeted searches of PubMed/MEDLINE, PubMed Central, publisher databases and citation trails up to June 2026.

Search terms and term combinations included myokines, skeletal muscle secretome, exercise, contraction, muscle–organ crosstalk, inter-organ crosstalk, extracellular vesicles, exerkines, IL-6, irisin, myostatin, follistatin, decorin, BDNF, FGF21, apelin, sarcopenia, osteoporosis, type 2 diabetes, obesity, COPD, cardiovascular disease, chronic inflammation, cancer cachexia and exercise medicine. Citation chaining was used to identify foundational mechanistic studies and recent translational reviews that were not captured by single keyword combinations. Given the conceptual objective of the review, priority was assigned to studies providing mechanistic insight, source attribution, temporal characterization of exercise-responsive signaling, or clinically relevant interpretation of muscle–organ communication. Recent high-impact reviews, consensus statements, translational studies and multi-omics investigations were preferentially included when they contributed directly to the development of the adaptome framework.

Studies were prioritized when they met at least one of the following criteria: direct relevance to skeletal muscle secretion; evidence of exercise responsiveness; mechanistic linkage to intracellular signaling pathways; demonstration of autocrine, paracrine or endocrine effects; relevance to human exercise physiology; or disease-focused interpretation of muscle–organ crosstalk. Human exercise trials and clinical rehabilitation studies were emphasized when available, whereas cell culture and animal studies were included when they clarified causal mechanisms that are difficult to isolate in humans.

Studies were de-emphasized when they measured circulating exercise-responsive molecules without plausible source attribution, when they used poorly characterized assays without methodological caution, when they focused on non-muscle tissues without relevance to muscle–organ communication, or when they supported broad therapeutic claims without functional outcomes. Because this review performs conceptual synthesis rather than effect-size estimation, no pooled estimates were calculated and no formal risk-of-bias scoring was performed.

To reduce conceptual ambiguity, the evidence was organized into three categories: bona fide myokines released by muscle cells; myokine-associated exerkines whose circulating concentration is exercise-responsive but not necessarily muscle-exclusive; and extracellular-vesicle-associated cargo that may participate in muscle–organ communication. Claims about therapeutic relevance were interpreted as strongest when four elements were present together: muscle origin, exercise responsiveness, receptor or target-tissue engagement and a measurable functional effect.

Because definitive source attribution is rarely available for every circulating factor in human exercise studies, we applied a graded source-confidence logic rather than a binary muscle/non-muscle classification. Signals were interpreted as confirmed muscle-derived when supported by biopsy, arteriovenous balance, cell-specific experimental models or tissue-specific vesicle capture; probable muscle-associated when supported by convergent muscle expression and exercise responsiveness; exercise-responsive but non-exclusive when multiple tissues could plausibly contribute; and unassigned when source evidence was insufficient. This grading was used to avoid treating circulating change alone as proof of myokine origin.

Study selection and evidence synthesis were performed iteratively by the authors, with emphasis on conceptual coherence, mechanistic plausibility and translational relevance to the proposed adaptome framework.

## 3. Skeletal Muscle as a Secretory and Signal-Integrating Organ

The muscle secretory environment is not generated by myofibers alone. Mature fibers produce contraction-responsive molecules, but the in vivo muscle niche also includes satellite cells, fibro-adipogenic progenitors, endothelial cells, pericytes, motor neurons, Schwann cells, macrophages and extracellular-matrix compartments. These cellular sources can amplify, buffer or reinterpret the secretory response to exercise and disease.

This distinction matters because the measured secretory signal reflects both myofiber activity and the condition of the muscle microenvironment. In healthy muscle, repeated exercise bouts tend to produce transient and coordinated secretory pulses. In aged, insulin-resistant, inflamed or immobilized muscle, the same contractile stimulus may occur against a background of mitochondrial dysfunction, extracellular-matrix stiffness, capillary rarefaction, immune-cell infiltration or anabolic resistance. The circulating myokine pattern may therefore indicate not only contraction, but also the readiness of the tissue to respond adaptively.

For this reason, the myokine adaptome should be distinguished from related but non-identical terms. The muscle secretome refers to the total set of molecules released by muscle or muscle-resident cells. Exerkines refer more broadly to exercise-responsive circulating factors, regardless of tissue origin. The myokine adaptome, as used here, refers to the adaptive signaling state generated when intracellular exercise inputs are converted into coordinated secretory patterns and interpreted by recipient tissues. It therefore includes source, timing, composition, delivery route and target-organ sensitivity rather than molecular identity alone.

Myokines can be delivered through classical endoplasmic reticulum (ER)–Golgi secretion, non-classical release mechanisms and vesicle-associated transport. Extracellular vesicles are especially relevant because they can carry proteins, lipids, mRNAs and microRNAs, thereby allowing skeletal muscle and other exercise-responsive tissues to communicate through packaged molecular information rather than isolated soluble ligands [18,19,20].

A major caution is that circulating extracellular vesicles after exercise are heterogeneous. Immune, endothelial, platelet, adipose and hepatic sources may contribute substantially to the total plasma vesicle pool. Exercise-induced extracellular-vesicle cargo should therefore not automatically be interpreted as muscle-derived unless tissue-specific markers, capture strategies, muscle biopsy correlations or experimental models support that conclusion [18,19,20].

## 4. Cellular Triggers of Myokine Release

Energetic stress is one of the most powerful triggers of exercise-induced signaling. During contraction, ATP hydrolysis increases AMP and ADP availability, accelerates mitochondrial respiration and changes substrate flux. AMPK functions as a cellular energy sensor and interacts with PGC-1α, SIRT1 and mitochondrial regulatory networks to promote oxidative remodeling, glucose transport, lipid oxidation and secretory adaptation [21,22].

Resistance exercise and eccentric loading impose a distinct adaptive demand: the muscle must sense tension, microdamage, matrix strain and growth requirements. Mechanical loading activates integrins, focal adhesion signaling, mechanosensitive channels and mTORC1-related anabolic pathways. This context favors signals involved in hypertrophy, remodeling, satellite-cell activation and extracellular-matrix turnover [23,24,25,26,27].

Calcium flux during excitation–contraction coupling activates CaMK, calcineurin and downstream transcriptional programs that influence fiber phenotype and mitochondrial adaptation. Redox signaling adds another layer. Contracting muscle produces reactive oxygen species that can function as physiological messengers, whereas chronic redox imbalance can damage proteins, impair force production and promote inflammatory signaling [28,29].

The IL-6 response illustrates the difference between adaptive and maladaptive interpretation. Acute exercise-induced IL-6 is typically transient and may contribute to glucose homeostasis, lipolysis and anti-inflammatory cascades. Chronically elevated IL-6 in obesity, aging or inflammatory disease reflects a different biological state and is often associated with insulin resistance, catabolism and systemic inflammation [30,31,32,33].

**Figure 1:**
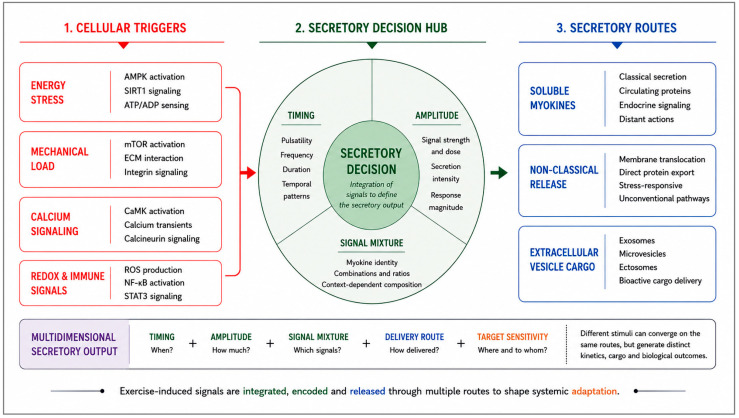
Cellular triggers and secretory routes of exercise-induced myokines. Energetic stress, mechanical loading, calcium signaling, and redox–immune pathways converge within a secretory decision hub that integrates signal timing, amplitude, and composition. This multidimensional encoding process determines the relative contribution of classical secretion, non-classical release, and extracellular-vesicle-mediated communication, thereby shaping the kinetics, cargo profile, and biological actions of exercise-induced signaling. Conceptual synthesis informed by energetic-stress, mechanotransduction, redox and autophagy/exerkine literature [12,21,22,23,24,25,28,29,34,35,36,37,38,39,40,41].

The intracellular inputs and secretory routes can be interpreted as an encoding layer, as illustrated in Figure 1.

## 5. Functional Classes of Myokines and Myokine-Associated Exerkines

Immunometabolic myokines connect contraction to substrate mobilization and immune regulation. IL-6 remains the prototype. Human studies established that contracting skeletal muscle can contribute substantially to the exercise-induced rise in circulating IL-6, that the response is influenced by glycogen availability and that IL-6 can participate in glucose homeostasis during contraction [42,43,44].

The interpretation of IL-6 has evolved from a simple inflammatory cytokine model toward a context-specific energy allocation model. Acute muscle-derived IL-6 may support substrate availability and anti-inflammatory signaling, whereas chronic IL-6 elevation from immune and adipose sources may contribute to metabolic dysfunction. This duality has been described as a double-edged sword and remains essential for any serious discussion of myokines in health and disease [30,31,32,33].

IL-15 is another immunometabolic candidate because it has been linked to muscle–adipose crosstalk, immune regulation and metabolic homeostasis. IL-15 has been associated with natural killer and T-cell homeostasis, muscle–adipose communication, fat-mass regulation and mitochondrial or oxidative metabolic effects, although human exercise studies remain heterogeneous and causality is less secure than for IL-6 [45,46,47]. However, the evidence varies across populations and protocols; exercise responsiveness alone is not equivalent to proof of direct muscle origin, receptor engagement or target-organ causality [45,46].

Myostatin, a member of the TGF-β superfamily, is a central negative regulator of skeletal muscle mass. Genetic and experimental studies show that myostatin suppresses muscle growth, while myostatin inhibition or functional loss promotes muscular hypertrophy [48,49]. In disease contexts such as sarcopenia, cachexia and chronic inflammation, excessive catabolic signaling may contribute to muscle wasting and impaired tissue resilience. In sarcopenia, evidence for uniformly elevated circulating myostatin is mixed; however, aging, disuse, cachexia and chronic inflammation can shift the remodeling environment toward greater myostatin/activin-SMAD catabolic tone relative to anabolic repair [50,51,52,53].

Follistatin, decorin and related extracellular-matrix mediators can counterbalance myostatin/activin signaling. Activins are TGF-β-family ligands that signal mainly through activin type II receptors and SMAD2/3 pathways, thereby promoting protein catabolism and opposing muscle growth in several wasting contexts [52,53]. Decorin is contraction responsive and has been implicated in resistance-training-related hypertrophic signaling, partly through interaction with myostatin pathways [54]. Meta-analytic evidence also suggests that resistance training can modulate circulating myostatin and follistatin, although heterogeneity across protocols and populations remains substantial [26]. Broader morphology-focused synthesis places these mediators within the larger hypertrophy-remodeling continuum [55].

Several myokines and exerkines connect muscle activity to adipose tissue, hepatic metabolism and systemic fuel handling. FNDC5/irisin attracted major attention after being proposed as a PGC-1α-dependent signal capable of promoting brown-fat-like thermogenic features in white adipose tissue [56]. Subsequent analytical work supported the detection of circulating human irisin by tandem mass spectrometry, while also emphasizing the need for rigorous assay validation [57]. Irisin research has therefore been most informative when interpreted across tissues: experimental studies link FNDC5/irisin signaling to adipose browning, bone remodeling and hippocampal BDNF-related neuroplasticity, whereas human translation depends strongly on assay specificity, sampling timing and functional endpoints [56,57,58,59,60,61].

Myonectin/CTRP15 has been described as a muscle-derived factor linking skeletal muscle to systemic lipid homeostasis [62]. Meteorin-like and β-aminoisobutyric acid have also been implicated in adipose tissue browning, lipid oxidation and cardiometabolic risk modification [63,64]. FGF21, although not muscle-exclusive, can increase after exercise and participates in metabolic stress responses involving liver, adipose tissue and muscle [65,66].

The muscle–brain axis is mediated by several exercise-responsive signals, including BDNF, irisin/FNDC5-related pathways and cathepsin B. Skeletal muscle cells can produce BDNF in response to contraction, and BDNF has been linked to AMPK-mediated fat oxidation in muscle [67]. Exercise-induced PGC-1α/FNDC5 signaling has also been connected to hippocampal BDNF expression in experimental models [58], while cathepsin B has been associated with running-induced systemic signaling and memory function [68]. Circulating irisin has also been implicated in exercise-linked cognitive regulation [59]. BDNF is relevant because it supports synaptic plasticity, learning and memory, and exercise-related BDNF signaling may arise from combined peripheral, central and vascular mechanisms rather than from skeletal muscle alone [58,67,68,69,70,71]. Accordingly, circulating BDNF should not be interpreted as a direct surrogate of skeletal muscle secretion, because platelets and the central nervous system are major contributors to systemic BDNF concentrations.

The muscle–bone axis is equally important. SPARC has been linked to exercise-related tumor suppression and tissue remodeling [72], and irisin has been reported to increase cortical bone mass in experimental models [60]. More broadly, muscle, bone and fat interact through mechanical loading and endocrine communication involving myokines, osteokines and adipokines [73].

Extracellular-vesicle-associated cargo represents a functional class rather than a single myokine. Exercise-responsive vesicles may contain proteins, lipids, mRNAs and microRNAs, and plasma miRNA signatures may also change after acute exercise and training [18,19,20,74]. In the adaptome framework, extracellular vesicles are important because they may encode signal combinations and temporal information that soluble single-molecule assays miss.

At the same time, extracellular-vesicle interpretation requires methodological discipline. Changes in circulating vesicle number or cargo after exercise do not automatically prove skeletal muscle origin. Future studies should combine plasma vesicle profiling with muscle tissue markers, cell-specific capture approaches, biopsy-linked transcriptomics or experimental models that can separate muscle-derived cargo from immune, endothelial, platelet, hepatic and adipose contributions.

The representative mediators in Table 1 were selected to separate bona fide myokines from broader exercise-responsive exerkines and EV-associated cargo.

It should be emphasized that the representative mediators summarized in Table 1 do not share the same level of mechanistic or clinical evidence. While IL-6 and myostatin are supported by extensive experimental and translational data, molecules such as BAIBA, meteorin-like, and myonectin currently remain supported by comparatively more limited human evidence. Accordingly, Table 1 should be interpreted as a conceptual overview rather than a ranking of evidential strength.

## 6. Muscle–Organ Crosstalk in Health

Muscle–organ crosstalk is the physiological space in which myokines become clinically meaningful. The muscle–adipose axis links exercise to lipolysis, adipose inflammation, thermogenesis and insulin sensitivity. IL-6, irisin, IL-15, myonectin, meteorin-like and BAIBA all contribute to this conceptual axis, although with different levels of evidence for direct muscle origin and target-tissue causality [81].

The muscle–liver axis is central to exercise-related glucose and lipid regulation. During and after exercise, muscle-derived signals interact with hepatic glucose production, lipid handling, ketone-related metabolism and inflammatory tone. FGF21, IL-6 and myonectin provide examples of factors through which contracting muscle may influence liver–adipose–muscle nutrient coordination [65,66,82,83,84,85,86].

The muscle–pancreas axis is less mature but important. Improved skeletal muscle glucose uptake reduces pancreatic insulin demand, while exercise-responsive mediators and EV cargo may influence β-cell function, inflammation and metabolic resilience. This axis illustrates a recurring theme: myokines rarely act alone; their effects are embedded in whole-body changes in substrate flux, insulin sensitivity, vascular perfusion and immune regulation [18,19,20,82,83,84,85,86].

The muscle–bone unit combines mechanical and endocrine communication. Loading improves bone through strain-dependent mechanisms, but muscle-derived factors such as irisin, myostatin-related pathways, SPARC and decorin may also influence osteoblast and osteoclast biology. Osteosarcopenia can therefore be interpreted as a breakdown of both mechanical loading and endocrine-muscle–bone crosstalk [60,72,73,87].

The muscle–brain axis is one of the most attractive areas for translational research. Exercise improves cognition and mood through vascular, inflammatory, metabolic and neurotrophic mechanisms, and muscle-derived factors may contribute to these effects. However, the brain is not a passive target. Local production of neurotrophic factors, blood–brain barrier dynamics and neural activity determine how peripheral signals are interpreted [69].

## 7. Myokine Dysregulation in Disease

The disease examples below are used as stress tests of the adaptome framework rather than as exhaustive reviews of each condition. They are included only to illustrate how different disorders can distort distinct parts of the communication loop: signal generation, vascular delivery, inflammatory background, tissue decoding and functional adaptation.

Disease does not merely reduce myokine secretion. It changes the timing, amplitude, composition and target-organ interpretation of the myokine signal. In obesity and type 2 diabetes, skeletal muscle insulin resistance, mitochondrial stress, adipose inflammation and ectopic lipid accumulation can reshape the muscle secretome and reduce the beneficial decoding of exercise-induced signals [82,83,84,85,86].

In these metabolic disease states, the same circulating mediator may carry different information depending on source and timing. A transient post-exercise cytokine pulse can reflect substrate mobilization and inflammatory resolution, whereas chronic low-grade cytokine elevation may indicate adipose–immune activation, impaired metabolic flexibility and reduced target-tissue responsiveness. The adaptome model therefore treats metabolic disease as a disorder of signal generation and signal interpretation, not only as a disorder of single circulating concentrations.

Sarcopenia and aging provide another example of adaptome remodeling. The revised European consensus defines sarcopenia through low muscle strength, low muscle quantity or quality and poor physical performance, emphasizing functional decline as much as tissue loss [50]. In this context, age-associated inflammation, neuromuscular impairment, satellite-cell dysfunction, mitochondrial decline and anabolic resistance may blunt the adaptive secretory response. Apelin has been proposed as an exercise-related factor capable of reversing aspects of age-associated sarcopenia in experimental work [88]. Apelin is induced by physical activity and has been linked experimentally to improved mitochondrial function, autophagy, myogenesis and metabolic resilience, making it a candidate mediator of exercise-related protection against age-associated muscle decline [88].

Osteoporosis and osteosarcopenia reflect disrupted muscle–bone communication. Reduced loading decreases mechanical stimulation, while altered myostatin, irisin, decorin, SPARC and extracellular-vesicle-mediated signals may impair bone remodeling. This is especially relevant for older adults, immobilized patients and chronic disease populations in whom muscle loss and bone fragility develop in parallel [73].

Cardiovascular disease adds a vascular decoding problem to the adaptome. Endothelial dysfunction, reduced capillary recruitment, chronic inflammation and impaired mitochondrial function may limit both the delivery of muscle-derived signals and the responsiveness of recipient tissues. Exercise training can improve vascular function, oxidative capacity and cardiometabolic risk, but the myokine response in this context should be interpreted together with perfusion, endothelial health, autonomic regulation and functional capacity rather than as an isolated endocrine readout [11,89].

COPD is strategically important for this topic because it combines systemic inflammation, peripheral muscle dysfunction, exercise intolerance and rehabilitation responsiveness. Official statements recognize limb muscle dysfunction as a major extrapulmonary feature of COPD, and pulmonary rehabilitation is an established intervention to improve exercise capacity and symptoms [90,91,92]. A myokine adaptome perspective may help explain how local muscle dysfunction and systemic inflammation interact with lung disease, inactivity and rehabilitation response [87].

Cancer and cachexia represent high-stakes contexts in which the muscle secretome may be both a victim and a mediator of disease. Tumor-driven inflammation, metabolic stress, reduced nutrient intake and treatment toxicity promote muscle wasting, while altered myostatin/activin signaling, inflammatory cytokines and extracellular-vesicle communication may reinforce catabolic remodeling. Exercise-induced myokines and extracellular vesicles have been investigated as potential mediators of improved treatment tolerance, inflammation resolution and altered tumor biology, but cancer-specific claims require caution because tumor type, disease stage, treatment phase and exercise prescription strongly modify interpretation [20,93,94].

Across these conditions, the central principle is that disease modifies both sides of the communication loop. The contracting muscle may generate a weaker, delayed, exaggerated or compositionally distorted signal, while recipient tissues may lose sensitivity through receptor changes, inflammation, vascular dysfunction or metabolic inflexibility. This dual impairment helps explain why the same exercise protocol can produce different molecular and clinical outcomes across populations.

The disease-specific remodeling patterns summarized in Table 2 synthesize evidence from metabolic disease, aging, sarcopenia, COPD, cardiovascular disease and cancer/cachexia literature.

**Figure 2:**
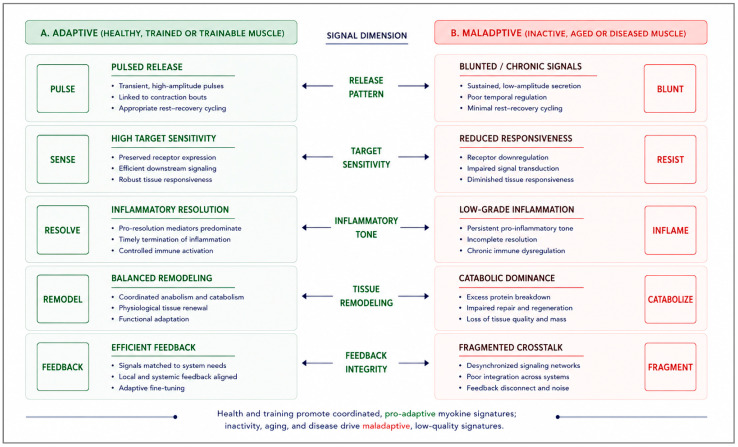
Adaptive versus maladaptive myokine signatures. Healthy trained muscle generates transient, coordinated and efficiently decoded signaling patterns, whereas aging and chronic disease may produce blunted, chronically elevated or compositionally distorted signals accompanied by impaired target-organ responsiveness. Conceptual synthesis informed by immunometabolic, aging, sarcopenia, cardiopulmonary and cachexia evidence [30,31,32,33,50,51,52,53,82,83,84,85,86,87,88,89,90,91,92,93,94,95,96,97,98,99,100,101,102,103,104,105,106,107,108,109,110,111,112,113,114,115,116,117].

The adaptive-versus-maladaptive interpretation can be operationalized as a contrast between pulse quality (defined here as appropriate amplitude, temporal coordination, return to baseline and compatibility with downstream functional improvement), target-organ sensitivity and inflammatory resolution, as illustrated in Figure 2.

## 8. Exercise as a Modulator of the Myokine Adaptome

Exercise is the most physiological modulator of the myokine adaptome. Acute exercise produces transient pulses, whereas chronic training reorganizes the baseline capacity to generate, deliver and decode those pulses. A useful distinction is therefore pulse versus remodeling: the former describes the immediate molecular response after a session; the latter describes the longer-term restructuring of muscle, vascular, immune and metabolic systems.

Endurance exercise primarily stresses energetic and mitochondrial pathways. It strongly engages AMPK-PGC-1α signaling, substrate mobilization and oxidative remodeling [21,24,25,34,35]. Resistance exercise primarily stresses mechanical and anabolic pathways, including mTORC1, extracellular-matrix remodeling and myostatin–follistatin–decorin balance [23,26,27,54]. High-intensity interval exercise combines energetic stress, calcium flux, lactate/pH shifts and vascular demand, potentially generating robust but protocol-sensitive secretory patterns [111,112].

Clinical interpretation requires attention to modifiers. Age, sex, training status, nutritional state, obesity, medication, sleep, circadian timing, disease severity and baseline inflammation can all change the myokine response to the same exercise protocol [13,14,15,103,104,118,119,120,121,122,123,124,125,126]. This helps explain why studies often report inconsistent responses for individual molecules. The adaptome model predicts heterogeneity; it does not treat it as noise.

### 8.1. Sex-Specific Modulation of the Myokine Adaptome

Emerging evidence suggests that biological sex may influence both the generation and decoding of exercise-induced myokine signals [118,119]. Differences in sex hormones, body composition, substrate utilization, inflammatory regulation and muscle fiber characteristics can alter the amplitude, timing and molecular composition of exercise-responsive signaling patterns [119,120,121,122]. Estrogen-related effects on mitochondrial function, oxidative stress handling and inflammatory regulation may influence the adaptive interpretation of exercise-derived signals, whereas androgenic signaling may modify anabolic–catabolic balance and remodeling responses [118,121,122]. Estrogen signaling may modulate myokine biology by supporting mitochondrial oxidative capacity, antioxidant defense and inflammatory resolution, which can alter both secretion kinetics and target-tissue interpretation. Androgen signaling may influence the anabolic–catabolic balance by interacting with mTOR-related remodeling, satellite-cell activity and myostatin/activin regulation, thereby shaping resistance-training-associated secretory profiles [118,121,122].

From the adaptome perspective, sex should not be viewed merely as a demographic covariate but as a biological modifier of signal encoding and target-organ sensitivity [118,119]. Two individuals exposed to identical exercise stimuli may therefore generate distinct signaling trajectories because hormonal milieu, receptor expression and tissue responsiveness differ. Future time-resolved studies should incorporate sex-stratified analyses to determine whether adaptive pulsatility, signal resolution and target-organ decoding follow sex-specific patterns across health, aging and chronic disease [118,119,122].

### 8.2. Research-Oriented Exercise-Prescription Implications

These implications should be read as a research-oriented prescription logic rather than as a validated clinical algorithm. In practical terms, the myokine response to exercise should be interpreted as an interaction between FITT variables (frequency, intensity, time and type) and biological context. For metabolic disease, a prescription may prioritize insulin sensitivity, mitochondrial flexibility and inflammatory resolution. For sarcopenia, it may prioritize anabolic remodeling, neuromuscular recruitment and anti-catabolic signaling. For COPD, it may prioritize peripheral muscle oxidative capacity, dyspnea tolerance and rehabilitation adherence.

This interpretation also cautions against universal “myokine-boosting” claims. A desirable response is not simply a higher concentration of a given mediator, but a signal pattern that is appropriately timed, resolved after the stimulus and decoded by the relevant target organs. Exercise prescription should therefore aim to restore adaptive pulsatility and tissue responsiveness, not only to increase circulating markers.

## 9. The Myokine Adaptome Model

The myokine adaptome model proposes that muscle-derived signaling should be described along four dimensions: amplitude, temporal pattern, molecular composition and target-organ sensitivity. Amplitude refers to the magnitude of secretion or circulating change. Temporal pattern refers to onset, peak, duration and return to baseline. Molecular composition refers to the coordinated mixture of cytokines, growth factors, metabolites and vesicle cargo. Target-organ sensitivity refers to the ability of recipient tissues to detect, transport, internalize and respond to the signal.

The operational novelty of the adaptome lies in combining these dimensions as minimum interpretive requirements rather than treating them as optional descriptors. Under this framework, an exercise-responsive factor becomes adaptome-relevant only when the study specifies the stimulus, estimates source confidence, captures the response trajectory, places the mediator within a co-signal or delivery context, and links the pattern to target-tissue or functional outcomes. This combined requirement is what differentiates the framework from conventional secretome catalogs, broad exerkine lists and descriptive inter-organ crosstalk models.

**Figure 3:**
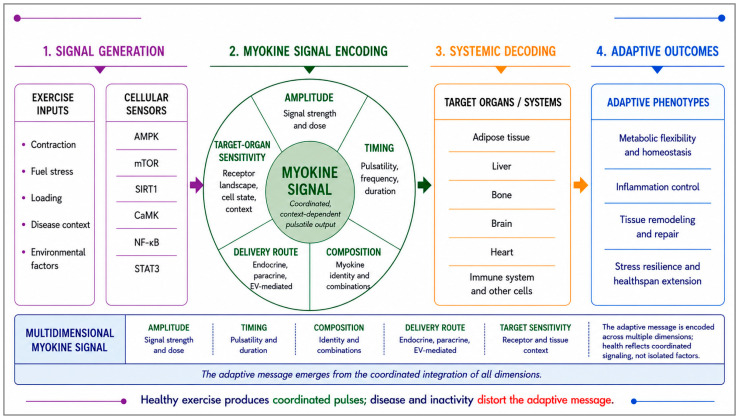
The myokine adaptome: from contractile stimuli to systemic adaptation. Exercise-derived inputs and contextual modifiers are integrated by intracellular sensing networks and encoded into multidimensional myokine signals defined by amplitude, timing, composition, delivery route, and target-organ sensitivity. These signals are decoded across multiple tissues and organs, ultimately shaping adaptive phenotypes related to metabolism, inflammation, remodeling, and resilience. Conceptual synthesis informed by systems physiology, molecular biomarker and multi-omics exercise literature [4,5,6,8,9,10,11,12,13,14,15,16,17,123].

The full generation–encoding–decoding–phenotype sequence is summarized in Figure 3, linking systems-level exercise physiology with source attribution and adaptive information patterns.

A central distinction of the present framework is that the myokine adaptome should not be viewed as a synonym for the muscle secretome. The muscle secretome describes the repertoire of molecules released by skeletal muscle and muscle-resident cells, whereas the adaptome describes the adaptive information encoded within those signals. In this view, biological meaning emerges not from the presence of a molecule alone but from the interaction among signal amplitude, temporal dynamics, molecular composition, delivery route and recipient-tissue responsiveness. Two individuals may therefore display similar circulating concentrations of a given mediator while exhibiting profoundly different adaptive outcomes because the broader signaling context differs. The adaptome is consequently defined by informational organization rather than molecular inventory [4,5,6,7,12,13,14,15,16,17,18,19,20,47,123,127,128,129,130,131].

The novelty of the model lies in shifting the analytical focus from molecules to adaptive information patterns. Rather than asking which mediators are present, the adaptome framework asks how biological information is encoded, transmitted and decoded across tissues during exercise, recovery, aging and disease. A mediator is not adaptive or maladaptive in isolation. Its interpretation depends on tissue source, exercise context, kinetics, co-released signals, receptor landscape, disease state and functional outcome.

In this sense, the adaptome differs from a secretome inventory: the secretome asks what is released, whereas the adaptome asks what pattern is produced, why it is produced, which tissue can decode it and whether the final response improves resilience.

To make the conceptual boundary and novelty claim explicit, Table 3 positions the myokine adaptome against related constructs. The key distinction is that the adaptome is not another inventory of released molecules, but a source-aware, time-resolved and function-linked interpretation of adaptive communication.

This model helps resolve apparent contradictions in the literature. A high IL-6 peak after acute exercise in a healthy person may be adaptive, whereas chronically elevated IL-6 in obesity may be maladaptive. A reduction in myostatin after resistance training may favor remodeling, whereas persistent myostatin signaling in cachexia may amplify wasting. A rise in irisin-like immunoreactivity may be biologically meaningful only if assay validity, tissue source and target response are established.

The model is also experimentally testable. A minimal adaptome study would combine standardized exercise stimuli with serial blood sampling, muscle or tissue-informed source attribution, validated assays, extracellular-vesicle characterization and functional endpoints such as strength, VO_2_max, insulin sensitivity, inflammatory status or patient-reported symptoms. More advanced designs would add proteomics, metabolomics, transcriptomics, receptor profiling and computational network analysis to distinguish secretion, circulation, delivery and biological decoding.

The most important implication is that disease progression may reflect loss of pulsed adaptive communication. In healthy systems, exercise creates temporary stress followed by resolution and overcompensation. In chronic disease, the system may remain trapped in a low-grade stress state with insufficient amplitude for adaptation, insufficient resolution for recovery or reduced target-organ capacity to decode beneficial signals.

Conceptually, the adaptome may be viewed as the communication layer that links exercise physiology to systems adaptation. Whereas the secretome describes the molecular repertoire of skeletal muscle signaling, the adaptome captures the organizational principles through which those signals acquire biological meaning. This distinction may help explain why identical molecules can be associated with divergent outcomes across health, aging and disease.

### 9.1. Conceptual Distinction Between the Myokine Adaptome and the Muscle Secretome

A central vulnerability of any new conceptual term is the possibility that it merely renames an existing construct. The myokine adaptome avoids this problem only if it is defined by information architecture rather than by molecular membership. In this stricter interpretation, the muscle secretome describes the vocabulary of released molecules, whereas the adaptome describes the grammar of adaptive communication: timing, amplitude, molecular combination, delivery route, source attribution and recipient-tissue decoding [4,5,6,7,12,13,14,15,16,17,47,123,127,128,129,130,131].

Practically, this means that a secretome analysis can stop at molecular identification, whereas an adaptome analysis cannot. It must test whether the same molecule behaves differently across timing, source-confidence category, co-released signals and recipient-tissue responsiveness. The framework therefore enables questions such as whether pulse resolution predicts functional improvement better than peak concentration, whether disease changes decoding despite similar circulating levels, and whether multi-marker trajectories outperform single-molecule readouts.

The distinction is therefore ontological rather than terminological. The secretome is defined by molecular membership, whereas the adaptome is defined by informational organization. In the secretome framework, biological interpretation begins with the identification of released molecules. In the adaptome framework, biological interpretation begins with the structure of the signal itself, including temporal dynamics, coordinated signal combinations, source attribution, delivery route and target-organ responsiveness. Thus, identical molecular repertoires may carry different biological meanings if their encoding–decoding architecture differs.

The adaptome can therefore be viewed as a biological information-processing framework analogous to communication systems in which signal generation, transmission and decoding collectively determine functional outcomes. In this interpretation, adaptive value emerges from coordinated signaling patterns rather than from the abundance of individual mediators. This distinction allows the framework to generate experimentally testable predictions concerning pulse quality, signal resolution, source specificity and recipient-tissue sensitivity that are not directly addressed by conventional secretome inventories. Consequently, the adaptome is not proposed as an alternative catalog of exercise-responsive molecules, but as a falsifiable framework for understanding how biological information is encoded, transmitted and interpreted across tissues during exercise, recovery, aging and disease.

This distinction has practical consequences. A secretome study can identify what muscle or muscle-resident cells release under a given condition. An adaptome study must additionally ask whether the signal is pulsed or chronic, whether it resolves after the stimulus, whether it is accompanied by compatible co-signals, whether it reaches the intended tissue and whether the target tissue remains biologically capable of responding [12,18,19,20,75,76,77,78,79,80,132].

The adaptome therefore reframes exercise-induced secretion as a context-sensitive communication system. A single mediator may be adaptive in one context and maladaptive in another. IL-6 after glycogen-depleted endurance exercise, myostatin suppression after resistance training and irisin-like responses after endurance training cannot be interpreted responsibly without timing, source, assay validity and downstream functional effects [13,23,24,25,26,30,31,32,33,34,35,36,42,43,44,56,57,132].

For this reason, the proposed framework should be used conservatively. It should not imply that all exercise-responsive molecules are myokines, that all myokines are therapeutic, or that a single circulating concentration can represent the systemic benefit of exercise. Its added value lies in forcing studies to measure the structure of the response rather than only the abundance of isolated mediators [4,5,6,7,17,37,38,39,40,41,47,61,70,71,127,128,129,130,131,132,133].

Importantly, a study does not become adaptome-based merely because it measures multiple myokines, includes serial sampling, or applies multi-omics approaches. The defining requirement is the explicit testing of whether source-confidence category, temporal structure, co-signal composition, delivery route, and target-organ responsiveness improve explanatory or predictive value beyond concentration-based secretome models. If these dimensions fail to provide incremental information, an adaptome interpretation is not justified. In this sense, the framework is intended to be falsifiable rather than universally applicable.

### 9.2. Testable Predictions of the Myokine Adaptome Model

To remain scientifically useful, the adaptome model must generate predictions that can be confirmed, refined or rejected. The predictions below translate the framework into experimentally testable expectations for standardized exercise perturbations, longitudinal training studies, disease-specific rehabilitation trials and multi-omics biomarker designs [12,13,14,15,16,17,23,24,25,34,35,36,41,51,75,76,77,78,79,80,103,104,123,124,125,126,132,134].

The eight core predictions are intentionally phrased around trajectories, source attribution, modality and functional outcomes, because the model predicts that adaptive value is carried by coordinated temporal patterns rather than by maximal elevation of any single molecule.

**P1.** Adaptive pulsatility. Healthy trained individuals should show larger but faster-resolving post-exercise myokine pulses than sedentary or chronically inflamed individuals. This can be tested with standardized endurance or HIIT bouts, serial sampling from baseline to 24 h recovery and stratification by functional status. The prediction would be weakened if pulse resolution and coordination do not differ after controlling for exercise dose and baseline inflammation.

**P2.** Disease-decoding deficit. Obesity, type 2 diabetes, COPD and sarcopenia should impair target-organ responsiveness even when circulating mediator concentrations change. Testing should pair plasma profiling with insulin sensitivity, vascular function, strength, VO_2_max, symptoms or receptor/uptake markers. The prediction would be weakened if clinical or tissue outcomes track mediator concentration alone without evidence of altered decoding.

**P3.** Modality-specific signatures. Endurance, resistance and HIIT should generate distinct signal configurations rather than uniform increases in single mediators. Cross-over trials with matched energy expenditure or workload, multi-marker panels and EV cargo profiling would directly test this claim. The prediction would be weakened if modalities produce indistinguishable profiles once workload is standardized.

**P4.** Recovery-quality trajectory. Adaptive responses should show timely return to baseline together with improved function, rather than persistent elevation. Serial recovery sampling after repeated training sessions should therefore be interpreted alongside performance and inflammatory outcomes. The prediction would be weakened if the highest chronic elevations consistently predict the best adaptation independently of recovery dynamics.

**P5.** Training remodeling. After 8–12 weeks of training, the same acute exercise challenge should produce a shorter, more coordinated and functionally efficient response. Before–after intervention designs are particularly suitable for this test. The prediction would be weakened if training improves function without any change in pulse coordination, timing or resolution.

**P6.** Multi-marker superiority. Panels combining soluble myokines, EV cargo, metabolites and clinical function should predict adaptation better than any single marker. Regression or machine learning models can compare single-marker and multi-layer prediction. The prediction would be weakened if single myokines consistently outperform integrated panels across cohorts.

**P7.** Source-attribution constraint. Only a subset of exercise-responsive circulating factors should correlate with muscle biopsy transcript/protein changes or tissue-specific EV capture. Concurrent biopsy, plasma proteomics and EV profiling are therefore essential. The prediction would be weakened if most circulating exerkines are strongly attributable to skeletal muscle without tissue-informed evidence.

**P8.** Maladaptive basal elevation. Chronically elevated basal inflammatory or catabolic signals should predict poor training response unless pulsatility and resolution improve. Rehabilitation studies in chronic disease can test this through baseline and serial follow-up measures. The prediction would be weakened if high basal inflammation or catabolic tone predicts superior adaptation without improved resolution.

Together, these predictions make the adaptome model falsifiable by linking molecular trajectories to source attribution, modality, recovery and functional outcomes [12,13,14,15,16,17,23,24,25,34,35,36,41,51,75,76,77,78,79,80,103,104,123,124,125,126,132].

### 9.3. Challenges, Falsification Risks and Source-Attribution Limits

The adaptome hypothesis also creates methodological risks. If used loosely, it could become a persuasive label for heterogeneous biomarker data. The framework is therefore strongest when its claims are restricted to patterns that are time-resolved, source-informed, assay-validated and connected to functional outcomes [39,40,41,51,52,53,75,76,77,78,79,80,95,96,97,98,99,100,101,102,103,104,105,106,107,108,109,110,114,115,116,117,135,136,137,138,139,140,141,142].

A realistic validation strategy should therefore treat source attribution as probabilistic and tiered. Definitive muscle origin is ideal but not always feasible; the minimum requirement is transparent classification of source confidence and explicit separation between confirmed myokines, muscle-associated exerkines and unassigned exercise-responsive factors.

The main challenges and safeguards are summarized in Table 4. These safeguards are not cosmetic; they determine whether the adaptome remains a testable systems model or collapses into a descriptive catalog of exercise-responsive molecules.

### 9.4. Unique Strengths, Boundaries and Validation Pathway

The model has three unique strengths in its strict form. First, it changes the unit of analysis from isolated mediator abundance to response architecture. Second, it forces source-confidence grading instead of assuming that circulating exercise-responsive molecules are muscle-derived. Third, it links molecular trajectories to recipient-tissue decoding and functional endpoints, making the framework useful only when it improves explanation or prediction beyond secretome, exerkine or single-marker approaches.

The model also has clear boundaries. It is not a clinical diagnostic tool, a universal biomarker panel, a claim that every exerkine is a myokine, or a proposal that exercise benefits can be replaced by one molecule. It should be used primarily to structure experimental design, interpret heterogeneous biomarker data and generate testable hypotheses until prospective validation is available.

Validation can proceed through a staged pathway: (1) standardized exercise perturbations with controlled nutrition, timing and participant phenotype; (2) serial sampling across baseline, immediate post-exercise, early recovery and longer recovery; (3) source-confidence assessment using muscle biopsy, arteriovenous balance, tissue markers, cell-specific extracellular-vesicle capture or experimental models where feasible; (4) multi-layer profiling of soluble factors, vesicle cargo, metabolites and clinical function; and (5) prospective testing of whether adaptome trajectories predict training response, rehabilitation outcomes or disease-specific functional improvement better than secretome-only or single-marker models.

Accordingly, the adaptome should be considered validated only if it provides incremental explanatory or predictive value. If pulse timing, source-confidence category, co-signal composition and target-organ decoding do not improve interpretation beyond existing approaches, the framework should be revised or rejected.

## 10. Biomarkers and Therapeutic Opportunities

Myokines are attractive biomarkers because they sit at the interface of muscle health, exercise dose and systemic disease biology. Potential applications include monitoring training responsiveness, identifying sarcopenic risk, stratifying cardiometabolic disease, evaluating pulmonary rehabilitation and tracking recovery from inflammatory or catabolic states. However, single-molecule biomarkers are unlikely to capture the complexity of the adaptome.

At present, these applications should be considered validation targets rather than ready-to-use clinical tests. The framework is intended to guide the design and interpretation of longitudinal exercise studies, not to provide immediate diagnostic thresholds or universal prescription rules.

A more realistic strategy is panel-based profiling expressed in prose and experimental design rather than as a fixed universal table. Such a panel would ideally combine immunometabolic mediators such as IL-6, anabolic–catabolic regulators such as myostatin, follistatin and decorin, metabolic stress signals such as FGF21, myonectin, β-aminoisobutyric acid and irisin/FNDC5-related measures, extracellular-vesicle cargo and conventional clinical outcomes. These molecular data should be interpreted together with strength, VO_2_max or exercise capacity, glucose–insulin indices, inflammatory markers, body composition and disease-specific symptoms.

Longitudinal sampling is essential because timing can change interpretation as much as molecular identity. A pre-exercise value, an immediate post-exercise peak, a 1 to 3 h recovery value and a longer recovery or training-adaptation measure may tell different stories. The clinically useful biomarker is therefore not simply a concentration, but a trajectory that reflects adaptive pulsatility, resolution and target-organ responsiveness.

**Figure 4:**
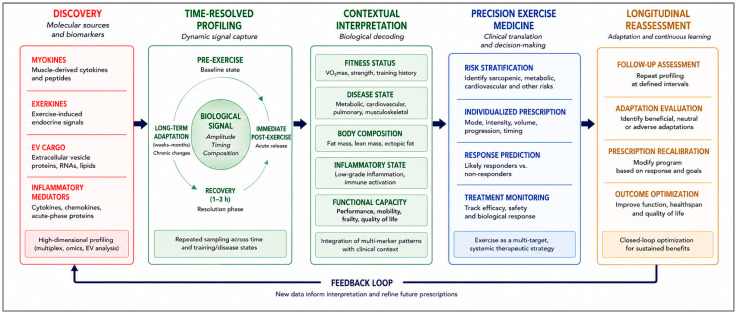
Translational pipeline from myokine biology to exercise medicine. Biomarker discovery and therapeutic translation require phenotyping, time-resolved profiling, mechanistic interpretation, individualized exercise prescription and longitudinal monitoring. Conceptual synthesis informed by exercise medicine, biomarker, immune-function and clinical exercise-prescription literature [89,101,102,103,104,110,113,116,117].

Taken together, these considerations support a shift from single-molecule assessment toward integrated, time-resolved and clinically contextualized evaluation of the myokine adaptome. The major translational steps linking mechanistic discovery to personalized exercise medicine are summarized in Figure 4. This translational logic is consistent with exercise-as-medicine, fitness-as-a-vital-sign and personalized exercise frameworks.

Therapeutic targeting is promising but difficult. Anti-myostatin approaches, FGF21 analogs, irisin-related pathways, apelin biology and extracellular-vesicle-based interventions have generated experimental and translational interest [48,49,56,57,60,62,63,64,65,66,88,94]. Yet the systemic benefits of exercise probably arise from coordinated network effects rather than from a single druggable mediator. This supports a cautious therapeutic philosophy: selected targets may be useful in specific diseases, but they should not be expected to reproduce the full biological complexity of exercise.

Exercise medicine remains the most powerful practical intervention. In its current form, the myokine adaptome model should be viewed as a framework for research-based prescription refinement rather than as a validated clinical decision tool. Rather than asking which myokine is good or bad, clinicians and researchers should ask which signal pattern is being produced, whether it is transient or chronic, which organs can respond and whether the pattern improves with training [89,113]. Selected examples of how this interpretive framework may influence clinical decision-making are presented in Box 1.

Box 1 **Potential clinical translation: how the adaptome framework may guide research-informed practice.**

- Obesity and type 2 diabetes: Prioritize restoration of metabolic flexibility, insulin sensitivity and inflammatory resolution rather than isolated cytokine suppression.
- Sarcopenia and aging: Monitor strength, muscle quality and anabolic–catabolic balance together with molecular pulse recovery.
- COPD and cardiovascular disease: Interpret myokine profiles alongside exercise tolerance, endothelial function, dyspnea burden and rehabilitation adherence.
- Cancer/cachexia: Treat molecular signals as context-dependent indicators of catabolic pressure, treatment phase and functional resilience rather than universal anti-tumor mediators.

## 11. Discussion

The myokine adaptome framework reframes exercise-induced muscle secretion as a context-dependent communication system rather than as a simple inventory of circulating mediators. Its central contribution is to integrate source attribution, secretion kinetics, molecular combinations, delivery route and target-organ responsiveness into a single interpretive structure. This is important because the same molecule can have different meanings depending on whether it appears as a transient post-exercise pulse, a chronic inflammatory signal or part of a broader disease-associated secretory profile.

This framework is therefore most useful when applied conservatively. It should not be used to imply that all exercise-responsive molecules are myokines, that all myokines are therapeutic, or that a single concentration can summarize the systemic benefit of exercise. The added value of the adaptome concept lies in its operational demand: studies should describe when a signal appears, where it comes from, which co-signals accompany it, which tissue can decode it and whether the final response improves function, resilience or disease status.

### 11.1. Alternative Explanations and Conceptual Challenges

A stronger version of the argument must also acknowledge alternative explanations. Some apparent adaptome patterns may reflect whole-body stress responses, hemodilution or hemoconcentration, immune-cell trafficking, platelet activation, hepatic or adipose release, or changes in clearance rather than direct skeletal-muscle secretion. Thus, a post-exercise rise in a mediator should not be equated automatically with a muscle-derived adaptive signal.

A second challenge is conceptual parsimony. The adaptome becomes scientifically useful only if it explains more than the muscle secretome, the exerkine response or generic inter-organ crosstalk. Its added value should therefore be judged by whether it improves prediction of functional adaptation, rehabilitation response or disease-specific decoding beyond single-marker or secretome-only models.

A third risk is therapeutic over-translation. Network-level exercise benefits should not be reduced to replacing training with one candidate molecule, EV cargo class or pharmacological mimic. The framework is most defensible when it guides study design and monitoring rather than when it is used to imply immediate druggability of complex exercise responses.

### 11.2. Future Research Priorities

Several limitations currently constrain the field. First, the definition of myokine remains inconsistent. Some molecules are clearly produced by contracting muscle, whereas others are exercise-responsive but also derived from liver, adipose tissue, immune cells, endothelium or bone. Second, many studies measure circulating concentrations without proving tissue source, secretion route, receptor engagement or causal target-organ effect. Third, assay variability remains a major issue, especially for molecules such as irisin/FNDC5-related readouts, but also for cytokines, extracellular-vesicle cargo and low-abundance proteins.

Timing is another major limitation. Single-time-point sampling can miss transient peaks, delayed responses, recovery failure or chronic basal elevation. Exercise protocols also differ widely in intensity, duration, recruited muscle mass, nutritional control, training status and circadian timing. These sources of heterogeneity are not merely methodological noise; they are biologically relevant modifiers of the adaptome response and should be incorporated into study design and statistical interpretation.

Disease states add a further layer of complexity. Age, sex, medication, obesity, inflammatory burden, renal function, sleep, nutrition and vascular access may alter both myokine production and clearance. Animal and cell models provide mechanistic depth, but they do not always reproduce human exercise physiology or clinical disease heterogeneity. A specific limitation of adaptome thinking is that it requires richer experimental designs than single-marker studies. Without serial sampling, tissue-informed source attribution, validated assays and functional outcomes, the model risks becoming a conceptual label rather than a measurable biological framework.

Future research should move toward time-resolved, tissue-informed and disease-specific mapping of the myokine adaptome. Single-cell and spatial transcriptomics can identify which muscle-resident cells contribute to secretory programs under exercise, aging and disease. Proteomics, metabolomics and extracellular-vesicle profiling can clarify how soluble and packaged signals differ across exercise modalities, while receptor and uptake profiling can help distinguish signal availability from biological decoding.

Human studies should prioritize repeated sampling before, during and after exercise, with standardized nutrition and careful phenotyping of training status, sex, age and disease severity. Where possible, muscle biopsies, plasma proteomics, extracellular-vesicle cargo, receptor expression and functional endpoints should be measured together. This design would allow researchers to distinguish secretion, circulation, delivery, tissue response and clinical adaptation rather than treating all circulating changes as equivalent.

Disease-specific studies are especially needed. Obesity, type 2 diabetes, sarcopenia, osteoporosis, cardiovascular disease, COPD and cancer/cachexia should not be treated as interchangeable chronic disease states. Each condition may distort a different part of the communication loop, including muscle signal generation, vascular delivery, inflammatory background, receptor expression, tissue repair capacity or functional adaptation. This disease-specific decoding is essential before adaptome profiles can be translated into rehabilitation monitoring or personalized exercise prescription.

Computational integration will become increasingly important. Machine learning and network models could identify multi-marker panels that predict adaptation, rehabilitation response or disease progression. However, these models must remain biologically interpretable. The goal is not merely to classify patients, but to understand how exercise changes communication between skeletal muscle and target organs, and why some individuals show adaptive remodeling whereas others display blunted, persistent or maladaptive signaling.

### 11.3. Clinical and Translational Implications

Clinically, the most realistic near-term application is not an exercise-mimetic replacement of training, but a better way to monitor whether exercise prescriptions restore adaptive pulsatility and target-organ responsiveness. A practical next step would be to design exercise studies around the five questions stated in the Introduction. Such studies would not only ask whether a molecule changes after exercise, but also when it changes, where it comes from, which signals accompany it, which tissue receives it and whether the person becomes stronger, more metabolically flexible, less inflamed or more functionally resilient.

Therefore, clinical translation should remain evidence-generating rather than prescriptive at this stage: adaptome profiles should inform validation studies, monitoring protocols and subgroup analyses before they are used for routine treatment decisions.

Several practical priorities follow from this translational logic. First, terminology should remain strict: bona fide myokines, broader exerkines and extracellular-vesicle-associated cargo should be separated explicitly. Second, temporal design is essential, because single sampling points may miss transient peaks, delayed responses or failed resolution. Third, source attribution should be strengthened through muscle biopsies, tissue markers, proteomic strategies, cell-specific extracellular-vesicle capture or arterio-venous sampling. Fourth, disease mapping should be stratified rather than generic, because obesity, type 2 diabetes, sarcopenia, osteoporosis, cardiovascular disease, COPD and cancer/cachexia may distort different parts of the communication loop. Finally, molecular readouts should be integrated with clinical outcomes such as strength, VO_2_max, exercise tolerance, body composition, inflammatory status and disease-specific symptoms, so that adaptome panels remain clinically useful rather than merely descriptive [12,13,14,15,16,17,23,24,25,27,34,35,36,39,40,41,51,52,53,75,76,77,78,79,80,95,96,97,98,99,100,101,102,103,104,105,106,107,108,109,110,114,115,116,117,123,124,125,126,132,135,136,137,138,139,140,141,142,143].

**Figure 5:**
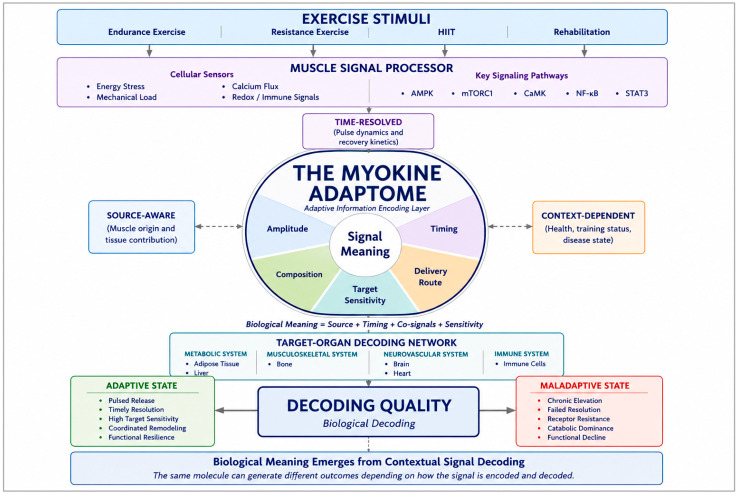
The myokine adaptome: an adaptive biological information-processing framework. Exercise stimuli are integrated by skeletal-muscle sensing networks and encoded into multidimensional signaling patterns defined by amplitude, timing, composition, delivery route and target sensitivity. The biological meaning of these signals is further shaped by source attribution, temporal dynamics and disease context before being decoded across metabolic, musculoskeletal, neurovascular and immune systems. Adaptive or maladaptive outcomes emerge from decoding quality rather than from the abundance of individual mediators alone, emphasizing that biological meaning is jointly determined by signal generation, contextual encoding and target-organ responsiveness.

Accordingly, the adaptome should be translated through standardized exercise perturbations, serial sampling, validated assays, tissue-informed attribution, multi-omics integration and clinically meaningful endpoints. Figure 5 condenses this logic into a final graphical synthesis linking exercise stimuli, muscle sensing, adaptome encoding, target-organ decoding and the emergence of adaptive or maladaptive outcomes through contextual signal interpretation.

## 12. Conclusions

Myokines occupy a central position in the biology linking skeletal muscle contraction to systemic health. They connect exercise to metabolism, inflammation, bone remodeling, neuroplasticity, vascular function, immunity and chronic disease prevention. Yet the field will advance more rapidly if myokines are not treated as isolated molecules with fixed meanings.

The myokine adaptome model proposed here frames skeletal muscle as a cellular signal processor that converts energetic, mechanical, redox, calcium-dependent and inflammatory information into coordinated secretory patterns. In health, these patterns are pulsed, adaptive and efficiently decoded by target organs. In disease, aging and inactivity, the pattern may become blunted, chronically elevated, compositionally distorted or poorly interpreted by recipient tissues.

For the Special Issue “Myokines in Health and Diseases”, this framework provides a comprehensive and mechanistic narrative linking cellular signaling to clinical translation. Its main contribution is to move the field from asking whether individual myokines rise or fall toward asking how exercise generates adaptive communication patterns, how disease distorts those patterns and how personalized exercise medicine can restore them. Future work should define disease-specific adaptome signatures, validate time-resolved biomarker panels and develop exercise prescriptions that rebuild coordinated muscle–organ communication.

Validation of this framework will require standardized exercise perturbations, serial sampling, source-aware multi-omics and clinically meaningful endpoints. MoTrPAC-style longitudinal designs, combined with disease-specific rehabilitation trials, can test whether restored pulse quality, signal resolution and target-organ decoding predict improvements in metabolic flexibility, function, symptoms and resilience. In this stricter form, the adaptome is not a new label for the secretome; it is a falsifiable translation framework for exercise medicine.

The framework’s originality therefore rests on testable operationalization and incremental predictive value, not on the novelty of any single component considered in isolation.

## Figures and Tables

**Table 1:** Major myokines and myokine-associated exerkines relevant to the myokine adaptome.

Mediator or Class	Exercise or Disease Context	Main Targets	Proposed Function	Interpretive Note
IL-6	Acute endurance or glycogen-depleted exercise; chronic inflammation	Liver, adipose tissue, immune cells, muscle	Fuel mobilization, glucose homeostasis, anti-inflammatory cascade [30,31,32,33,42,43,44,47]	Meaning depends strongly on timing and source
IL-15	Exercise-responsive immunometabolic signaling	Adipose tissue, immune cells, muscle	Muscle–adipose crosstalk and immune regulation [45,46,47]	Mechanistic certainty varies across protocols
Myostatin	Aging, disuse, cachexia; training modulation	Muscle, bone, connective tissue	Negative regulation of muscle growth [48,49,50,52,53]	Central catabolic/remodeling signal
Follistatin/decorin	Resistance exercise, ECM remodeling	Muscle fibers, ECM, myostatin pathways	Counter-regulation of myostatin and hypertrophic remodeling [26,54,55]	Relevant for resistance training adaptation
FNDC5/irisin	Endurance exercise, PGC-1α signaling	Adipose tissue, bone, brain, muscle	Thermogenic, bone and possible neuroplasticity signaling [56,57,58,59,60,61]	Requires rigorous assay validation
FGF21	Exercise and fasting-like metabolic stress	Liver, adipose tissue, muscle	Fuel stress response and lipid/glucose metabolism [65,66]	Not muscle-exclusive
Myonectin/CTRP15	Exercise and lipid flux	Liver, adipose tissue	Systemic lipid homeostasis [62]	Representative metabolic myokine
BDNF/cathepsin B	Exercise–neuroplasticity pathways	Brain, muscle and metabolic tissues	Fat oxidation, memory and neuroplasticity links [58,59,67,68,69,70,71]	Peripheral-to-brain causality remains complex
SPARC/irisin-bone axis	Exercise, ECM and bone contexts	Colon tissue, bone, muscle–bone unit	Tissue remodeling, bone effects and possible tumor-related effects [60,72,73]	Illustrates muscle–bone/tissue crosstalk
Extracellular-vesicle cargo	Acute exercise, chronic training, disease contexts	Multiple organs	Packaged proteins, lipids and RNAs [18,19,20,74,75,76,77,78,79,80]	Source attribution is essential

Note. Mediators are grouped by dominant interpretive role, not by exclusive tissue origin. Exercise responsiveness alone should not be read as proof of skeletal-muscle origin; source attribution and assay validity remain essential. Abbreviations: BDNF, brain-derived neurotrophic factor; CTRP15, C1q/tumor necrosis factor-related protein 15; ECM, extracellular matrix; FGF21, fibroblast growth factor 21; FNDC5, fibronectin type III domain-containing protein 5; IL, interleukin; PGC-1α, peroxisome proliferator-activated receptor gamma coactivator 1-alpha; RNA, ribonucleic acid; SPARC, secreted protein acidic and rich in cysteine.

**Table 2:** Disease-specific remodeling of the myokine adaptome.

Disease Context	Adaptome Distortion	Mechanistic Interpretation	Clinical Consequence	Exercise Relevance
Obesity/ type 2 diabetes	Inflammatory background and impaired insulin signaling	Adipokine–myokine imbalance and reduced target sensitivity [82,83,84,85,86,95,96,97,98]	Insulin resistance and low metabolic flexibility	Aerobic/resistance training may restore glucose uptake and responsiveness [89,99,100,101,102,103,104]
Sarcopenia/aging	Anabolic resistance and higher catabolic tone	Myostatin/activin dominance, mitochondrial decline, impaired regenerative niche [50,51,52,53,88]	Weakness, frailty and reduced resilience	Progressive resistance and multimodal exercise are central
Osteoporosis/ osteosarcopenia	Reduced loading plus altered muscle–bone endocrine communication	Impaired balance among irisin, SPARC, decorin and myostatin-related pathways [60,72,73,87]	Fracture risk and coupled muscle–bone decline	Resistance and impact-loading programs may improve crosstalk
Cardiovascular disease	Reduced vascular responsiveness and chronic inflammation	Endothelial dysfunction limits signal delivery and decoding [11,89,101,105]	Lower functional capacity and cardiometabolic risk	Aerobic and interval training improve vascular and mitochondrial profiles
COPD	Peripheral muscle dysfunction, systemic inflammation, inactivity cycle	Muscle–lung–immune crosstalk becomes maladaptive [87,90,91,92,106]	Exercise intolerance, dyspnea and reduced quality of life	Pulmonary rehabilitation can be framed as adaptome retraining [90,91,92]
Cancer/cachexia	Tumor-driven inflammation and catabolism	Muscle secretome may influence immune tone and tumor microenvironment [20,93,94,107,108,109,110]	Muscle loss, fatigue and reduced treatment tolerance	Exercise oncology requires disease-specific prescription [109,110]

Note. The table interprets disease as a distortion of both signal generation and signal decoding. The proposed patterns should be tested with standardized exercise stimuli, serial sampling and functional endpoints. Abbreviation: COPD, chronic obstructive pulmonary disease.

**Table 3:** Novelty and positioning of the myokine adaptome relative to related exercise-signaling concepts.

Existing Concept	Primary Focus	Main Limitation	Adaptome Perspective
Muscle secretome	Molecular repertoire released by skeletal muscle and muscle-resident cells.	Often interpreted as a static inventory or abundance profile.	Treats secretion as adaptive information defined by timing, source, composition and decoding.
Exerkines	Exercise-responsive circulating factors from muscle and non-muscle tissues.	Exercise responsiveness alone does not establish skeletal-muscle origin.	Separates bona fide myokines from broader exercise-responsive signals through source-aware interpretation.
Inter-organ crosstalk	Communication among muscle, adipose tissue, liver, bone, brain, immune and vascular systems.	Often describes organ links without a clear temporal encoding–decoding structure.	Adds a generation–encoding–decoding sequence and explicitly includes target-organ sensitivity.
Biomarker panels	Multi-marker prediction of training response, recovery or disease status.	Can remain descriptive if not connected to tissue source and function.	Requires serial sampling, validated assays, source attribution and clinically meaningful endpoints.
Myokine adaptome	Adaptive signaling state produced and decoded during exercise, recovery, aging and disease.	Still requires prospective validation in standardized human perturbation studies.	Provides a falsifiable framework for personalized exercise medicine and rehabilitation monitoring.

Note. The table clarifies why the adaptome is presented as an information-processing and decoding framework rather than as a renamed secretome. The distinction must be operationalized through timing, source attribution, multi-marker profiles and functional outcomes [4,5,6,7,12,13,14,15,16,17,18,19,20,47,127,128,129,130,131].

**Table 4:** Critical challenges, falsification risks and methodological safeguards for adaptome studies.

Challenge	Why It Threatens Interpretation	Recommended Safeguard
Source attribution	Circulating factors may originate from liver, adipose tissue, immune cells, endothelium, platelets or bone rather than skeletal muscle.	Use muscle biopsies, tissue-specific markers, cell-specific EV capture, arterio-venous sampling or experimental source models.
Timing heterogeneity	Single time points can miss transient peaks, delayed responses or resolution failure.	Use serial sampling before exercise, immediately after, during early recovery and after longer recovery/training periods.
Assay variability	ELISA kits, sample handling, serum/plasma choice and EV isolation methods can change apparent concentrations.	Use validated assays, report pre-analytics, include technical replicates and apply orthogonal confirmation where possible.
EV heterogeneity	Total plasma EV cargo is a mixed signal from multiple tissues and cell types.	Combine size/exclusion or immunocapture strategies with cargo validation and tissue-informed interpretation.
Causality versus association	Mediator changes may accompany exercise without driving functional adaptation.	Pair biomarker profiling with receptor engagement, pathway activation and functional endpoints.
Disease confounding	Age, obesity, medications, nutrition, sleep and inflammation can distort both secretion and clearance.	Stratify cohorts, standardize nutrition and medication timing where possible and model baseline inflammatory state.
Target-organ decoding	Adaptive meaning depends on receptor landscape, perfusion, uptake and tissue sensitivity.	Measure downstream tissue function, receptor/transport markers, vascular access or clinical response, not only circulating concentrations.

Note. These safeguards are designed to prevent overclaiming single-molecule causality. They draw on epigenetic, miRNA, EV, autophagy, metabolic disease, sarcopenia, cardiopulmonary, cancer/cachexia and personalized-exercise literature [39,40,41,51,52,53,75,76,77,78,79,80,95,96,97,98,99,100,101,102,103,104,105,106,107,108,109,110,114,115,116,117,135,136,137,138,139,140,141,142].

## Data Availability

No new data were created or analyzed in this study. Data sharing is not applicable to this article.

## Abbreviations

AMPK	AMP-activated protein kinase
BAIBA	β-aminoisobutyric acid
BDNF	brain-derived neurotrophic factor
CaMK	calcium/calmodulin-dependent protein kinase
COPD	chronic obstructive pulmonary disease
ECM	extracellular matrix
EVs	extracellular vesicles
FGF21	fibroblast growth factor 21
FITT	frequency, intensity, time and type
FNDC5	fibronectin type III domain-containing protein 5
GLUT4	glucose transporter type 4
HIIT	high-intensity interval training
IL-6	interleukin-6
IL-15	interleukin-15
miRNAs	microRNAs
mTOR	mechanistic target of rapamycin
mTORC1	mechanistic target of rapamycin complex 1
NF-κB	nuclear factor kappa B
PGC-1α	peroxisome proliferator-activated receptor gamma coactivator 1-alpha
ROS	reactive oxygen species
SIRT1	sirtuin 1
STAT3	signal transducer and activator of transcription 3
T2D	type 2 diabetes
TGF-β	transforming growth factor beta
VO_2_max	maximal oxygen uptake
